# Relative age effects in track-and-field: Identification and performance rebalancing

**DOI:** 10.3389/fphys.2022.1082174

**Published:** 2023-01-12

**Authors:** Audrey Difernand, Quentin De Larochelambert, Sébastien Homo, Florian Rousseau, Juliana Antero, Jean-François Toussaint, Adrien Sedeaud

**Affiliations:** ^1^ IRMES—URP 7329, Institut de Recherche Médicale et d’Epidémiologie du Sport, Université Paris Cité, Paris, France; ^2^ INSEP, Institut National du Sport, de l’Expertise et de la Performance, Paris, France; ^3^ Fédération Française d’Athlétisme, Paris, France; ^4^ Fédération Française de Cyclisme, Montigny-le-Bretonneux, France; ^5^ Centre d’Investigation en Médecine du Sport, Assistance Publique—Hôpitaux de Paris, Paris, France

**Keywords:** youth performance, talent identification, rebalancing method, corrective adjustment procedure, athlete development

## Abstract

**Introduction:** Relative Age Effect (RAE) consists of a biased distribution of the dates of birth in a same-age group.

**Objectives:** This study aimed to investigate Relative Age Effect among French athletes in different track-and-field events, and propose a corrective adjustment method to highlight the true potential of an athlete with respect to his/her relative age.

**Methods:** 358,610 performances from 2009 to 2019 of female and male athletes between 12 and 21 years old were collected. Relative age distributions of performances were analyzed by level of competitiveness (“All,” “Top50%,” “Top10%” where “all” represents all athletes, top50% and top10% represent the best 50% and 10% of athletes per age category respectively) and age category, with chi-square and odd-ratio statistics. A linear relationship between distribution of performances and age leads to a calibration coefficient allowing to rebalance the performance by considering the effect of Relative Age Effect. Validation is obtained by Wilcoxon statistical test on actual athlete data.

**Results:** Relative Age Effect is present in all types of events. It is larger when the level of competitiveness increases. In male 100 m sprint, 1 year difference between two athletes birth date represents an average gain of 931.01 ms (6.5%) in the U13 (Under 13 years old) and 229.65 ms (1.9%) in the U17 (Under 17 years old) categories. Our validated rebalancing methods allows to compensate for the biases induced by the relative age effect. By comparing the rebalanced performance and the realised performance of each athlete, we cannot say that they are significantly different. On average, there is no significant difference between these two performances.

**Conclusion:** This study showed that there is a relative age effect among young French athletes, with an even greater effect as the level of competition increases. Thanks to the rebalancing method that has been validated, performances can now be better appreciated according to category and event.

## Introduction

In sports, the relative age effect (RAE) is related to an over-representation of athletes born right after the cut-off date and an under-representation of those born just before the next cut-off date (Roberts et al., 2021). It shows a skewed distribution of athletes, and has been demonstrated among girls (Smith et al., 2018) and boys (Cobley et al., 2009)—with a smaller effect among females—and in team sports and individual sports (Cobley et al., 2009). Indeed, in track-and-field, U18 females and males born in January or February are respectively 2 and 3 times more likely to be ranked in the Top 100 best athletes than those born at the end of the year (Brustio et al., 2019). The relative age effect creates a bias on performances, whereby athletes born close to the cut-off date have an advantage compared to their peers born 12 months or 24 months later, depending on whether it is a one-year or a two-years category. In English track and field (Kearney et al., 2018) it is present in the majority of events and maximal in the U13 category—where U13 (*Under 13* *years old*) represents a two-year age category composed of young athletes, who are 11 or 12 years old). Although RAE seems greater at this age, one study investigated Italian long and high jump athletes between 15 and 20 years old (Boccia et al., 2017). Only 10%–25% of top level adults were already in the top level at age 16 (Boccia et al., 2017). It is possible that the RAE, with a feeling of discouragement (Hollings et al., 2014) generated by non-selection, the consecutive dropouts or cumulative disadvantages [better coaching, counterparts and competition (Wattie et al., 2008)] plays a role. RAE persists in track-and-field (Brustio and Boccia, 2021): it is greater among World Youth (U17) than in Junior Athletics Championships (Hollings et al., 2014), but decreases when sprinters get older (Romann and Cobley, 2015) or when competitiveness increases. The effect has also been observed in another metric sport which initiates the first theoretical readjustments of this effect: swimming (Cobley et al., 2019).

Solutions have already been settled to compensate for the bias induced by RAE: by focusing on organizational [e.g., rotating cut-off dates (Helsen et al., 1998) or classifying athletes by maturation status (Cumming et al., 2017; Cumming et al., 2018)] or practical strategies [e.g., shirt numbering based on month age (Mann and Ginneken, 2017) or correction factor to performance results (Abbott et al., 2021)]. Though performance does not progress linearly with age (Berthelot et al., 2012; Berthelot et al., 2019), corrective adjustments have been proposed with linear regressions in each age category (Romann and Cobley, 2015). Slope coefficients from linear regressions were used to rebalance performance (Cobley et al., 2019). Indeed, each day, week or month of difference between two athletes generates a difference in performance (Cobley et al., 2019). Quadratic equation models have been used to fit the best curve among sprinters performances: between 15 and 16.99 years old (U17), their compensating method was based on how far the age at performance was from 16 (Brustio and Boccia, 2021). The common goal was to reduce the RAE by birth quarters distribution. A recent study (Brustio et al., 2022) applied corrective adjustment procedures (CAPs) as a strategy to remove relative age effects on 689 junior Italian long jumpers. A recent study on French skiers showed that it is possible to cancel out the relative age effect through recalibration (De Larochelambert et al., 2022). Based on a regression coefficient per age category, performances are rebalanced according to their exact age and initial performance (De Larochelambert et al., 2022). Using the same methods, corrective adjustment procedure were applied to more objectively monitor the performance realized by each swimmer with respect to chronological age, in all events (Difernand et al.). With this recalibration method, swimmers’ performances were interpreted at the same exact age, in a more equitable way (Difernand et al.).

There is no study on the relative age effect on young French athletes to our knowledge, and even in the scientific literature, at the international level, the subject is not very explored. In the quest for top performances, records and medals at the international level, detection is the basis of this pathway (Williams et al., 2020). The better the detection, the better the high-level athletes. This is why we try to explore all possible aspects of detection, starting with the relative age effect (Cobley et al., 2009). Thus, our aim is to study the relative age effect in French Track and Field, and to propose a corrective adjustment method to better evaluate the potential of each athlete.

## Materials and methods

### Participants

The dataset was composed of French Track and Field athletes aged 12–22 and classified into one-year age categories, from U13 (Under 13) to U23 (Under 23). It included 189,238 male and 169,372 female performances, from athletes who competed at least once in local, regional, national or international competitions since 2009. All recorded performances conformed to standards of the International Association of Athletics Federations and were collected by the French Track and Field Federation. Date of birth, date of competition and all performances were collected. Also, the level of competitiveness was considered as follows: “All,” “Top50%” which corresponds to the best 50% of the age category in the event concerned and “Top10%” which corresponds to the best 10% in an equivalent way. For both male and female, events included 100, 200, 400, 800, 1,000, 400 m hurdles, shot put, discus throw, javelin throw, hammer throw, high jump, pole vault, long jump, triple jump plus 100 m (for female) or 110 m (male) hurdles. Other events were not considered due to lack of data in youth categories.

### Analysis

All statistical analyses were performed using Python (version 3.8.5; Python Software Foundation, Delaware, United States).

#### Part 1: Presence of RAE

In this first part, all athletes were considered. For each of them, only the best performance was kept. Then, athletes were divided into birth quarters according to their month of birth (Q1: January, February, March, Q2: April, May, June, Q3: July, August, September, Q4: October, November, December). In order to highlight the existence of RAE within the real age categories, birth quarters distributions were compared to the French distribution of births covering the birth years of all athletes in the study (French Institute of Statistics and Economic Studies). Indeed, the population was distributed as follows: 178,080 (23.64%) in Q1, 185,727 (24.65%) in Q2, 198,872 (26.40%) in Q3 and 190,702 (25.31%) in Q4. A chi-square test was used to determine whether the proportions of the population are consistent with the theory, in this case the French population born on the same years.

#### Part 2: Rebalancing methods

In this section, athletes who were actually involved in the discipline were included, only if they performed at least 6 times over the 6 years considered, between the ages of 12 and 17. Athlete’s exact age was obtained by calculating the difference between the date of birth and the competition date. Relative age gap was defined by the time delay between athlete’s last birthday and competition date, as it can be interpreted as an index to quantify athlete’s experience within his/her age category. Using a method similar previously employed (De Larochelambert et al., 2022; Difernand et al.), linear regressions for each age group between the relative age gap and the average of performances were computed in order to obtain the slope value “*c*.” The slope coefficient was converted into days and used to calculate the recalibrated coefficient: r = c*d where “*c”* is the slope value and “*d”* the number of days until the next birthday. The recalibrated coefficient “*r*” was subtracted (in race events, measured in seconds) or added (in jumps or throws, measured in meters) to the performance in order to obtain rebalanced performances.

#### Part 3: Testing rebalancing methods

In order to validate our method, we kept for each athlete from the previous base, two performances of the same age category (T1 and T2) if they were separated by at least 6 months (to allow sufficient time for progression). The rebalancing method was applied to the first performance (T1) in order to obtain the rebalanced performance (T2′) using the following formula:

$$T2^{\prime }=T1-r*\left(dT2-dT1\right)$$

dT2: exact age of realized performance in days
dT1: exact age of first performance in days

In this part, our objective was to show that the second realized performance (T2) is not statistically different from the rebalanced performance (T2′) in order to validate the rebalancing method. After checking that the data did not follow a normal distribution with the Kolmogorov Smirnov test, we used a non-parametric Friedman test to see if the three types of performance followed the same distribution.

Therefore, as it was not the case, a Wilcoxon test was performed to identify which performance was statistically different from the other two. Thus, by performing the Wilcoxon test on each possible pair (T1-T2′, T1-T2, T2-T2′), it is possible to show that: if the first performance (T1) is statistically different from the other two, it can be concluded that the other two performances (T2 and T2′) were not statistically different. Thus, if there is no statistical difference between the rebalanced performance and the realized performance, this will validate the rebalancing method.

### Ethics

Our sample was obtained from the French Federation of track-and-field. All data were reported anonymously. This study was designed and monitored by the IRMES (Institut de Recherche bio-Médicale et d’Epidémiologie du Sport) scientific committee. Data collection was compliant with the General Data Protection Regulations applied in the European Union. The protocol was approved by the ethics panel of CSMF (Conseil Scientifique, Médical et de Formation) from INSEP (Institute National du Sport, de l’Expertise et de la Performance). The use of retrospective data obtained from public sources was also approved by the Commission Nationale de l’Informatique et des Libertés (CNIL No. 2223498), the governing office supervising data utilization in France.

## Results

### Part 1: Presence of RAE

**FIGURE 1:**
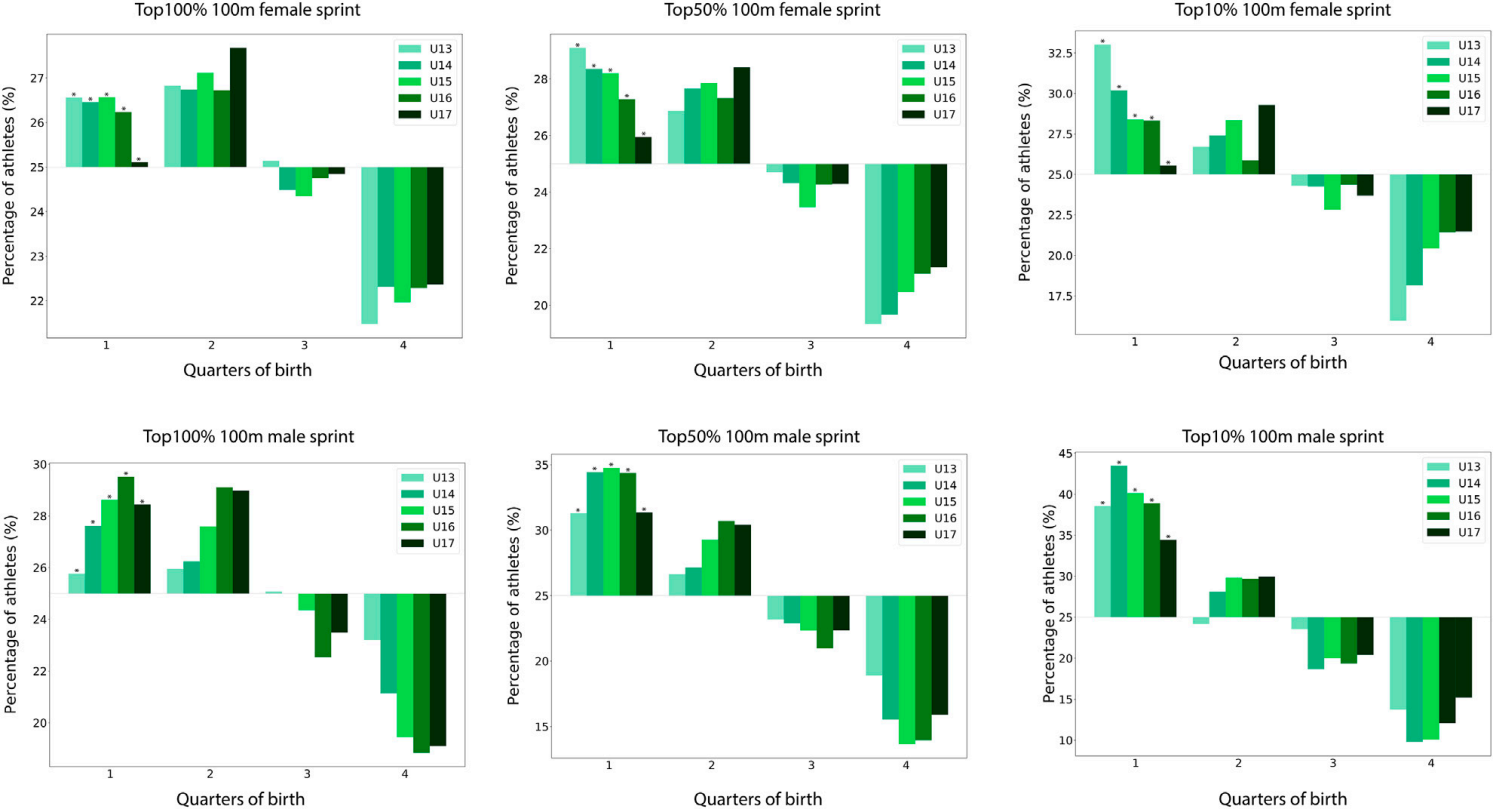
Birth quarters distribution according to age categories and performance level for female and male 100 m sprint. *: p < .01.

Birth quarters distribution on the youngest age categories for “All,” “Top50%,” and “Top10%” female and male athletes in 100 m sprint is presented in Figure 1. For any sex or performance level, not only the distribution of athletes’ birth quarters significantly differs from the French distribution of births, but also the proportion born in Q4 is lower than the proportion in any other quarter.

For U13 females, the proportion of 100 m sprinters born in Q4 is 21.4%, 19.3% and 16.2% for “All,” “Top50%,” and “Top10%” respectively. Similarly, among males, the proportion of Q1 increases considerably among U14: 27.6%, 34.4%, and 43.4% for “All,” “Top50%,” and “Top10%” respectively.

For males, the RAE is accentuated by the proportion of Q4 among U13 and U17, which are 23.2% and 19.1% respectively in the “All” category. Among the “Top50%” and “Top10%,” the effect is maximal in the U15 category and decreases thereafter. The distribution of birth quarters by age category for all events and sexes for “All” and “Top10%” is presented in the Supplementary Material, with an under-representation of athletes born in Q4 for the majority of events.

### Part 2: Rebalancing methods

After running linear regressions on performance as a function of the relative age gap, the resulting coefficients are presented for 100 m female and male sprint in Tables 1, 2 respectively. The coefficient is highest among the U13, regardless of sex. For females, within the same age group, there can be 564.1 ms (3.875%), 141.0 ms (1.0%), 47.0 ms (.3%) and 1.5 ms (.01%) of difference on average per year, quarter, month and day respectively. For males, the difference is greater, with a year, quarter, month and day generating an advantage of 931.0 ms (+6.5%), 232.8 ms (+1.6%), 77.6 ms (+.5%) and 2.6 ms (+.02%) respectively.

**TABLE 1:** Mean performance differences by year, quarter, month and day, among female 100 m sprinters, Δ, mean difference; ms, milliseconds; s, seconds; %, in percentage of performance mean.

Age	Mean (s)	Δ day in ms (%)	Δ month in ms (%)	Δ quarter in ms (%)	Δ year in s (%)
U13	14, 56	−1.54 (−.01)	−47.01 (−.32)	−141.04 (−.97)	−.564 (−3.87)
U14	14, 10	−1.25 (−.01)	−37.95 (−.27)	−113.86 (−.81)	−.455 (−3.23)
U15	13, 79	−.71 (−.01)	−21.67 (−.16)	−65.00 (−.47)	−.260 (−1.89)
U16	13, 55	−.75 (.01)	−22.75 (−.17)	−68.26 (−.50)	−.273 (−2.01)
U17	13, 37	−.34 (−.01)	−10.39 (−.08)	−31.16 (−.23)	−.124 (−.93)

**TABLE 2:** Mean performance differences by year, quarter, month and day, among male 100 m sprinters, Δ, mean difference; ms, milliseconds; s, seconds; %, in percentage of performance mean.

Age	Mean (s)	Δ day in ms (%)	Δ month in ms (%)	Δ quarter in ms (%)	Δ year in s (%)
U13	14, 33	−2.55 (−0.02)	−77.58 (−0.54)	−232.75 (−1.62)	−0.931 (−6.50)
U14	13, 42	−2.17 (−0.02)	−66.01 (−0.49)	−198.03(−1.48)	−0.792 (−5.90)
U15	12, 74	−1.75 (−0.01)	−53.18 (−0.42)	−159.55 (−1.25)	−0.638 (−5.01)
U16	12, 24	−1.29 (0.01)	−39.12 (−0.32)	−117.36 (−0.96)	−0.469 (−3.83)
U17	11, 93	−0.63 (−0.005)	−19.14 (−0.16)	−57.41 (−0.48)	−229.65 (−1.93)

### Part 3: Testing rebalancing methods

**FIGURE 2:**
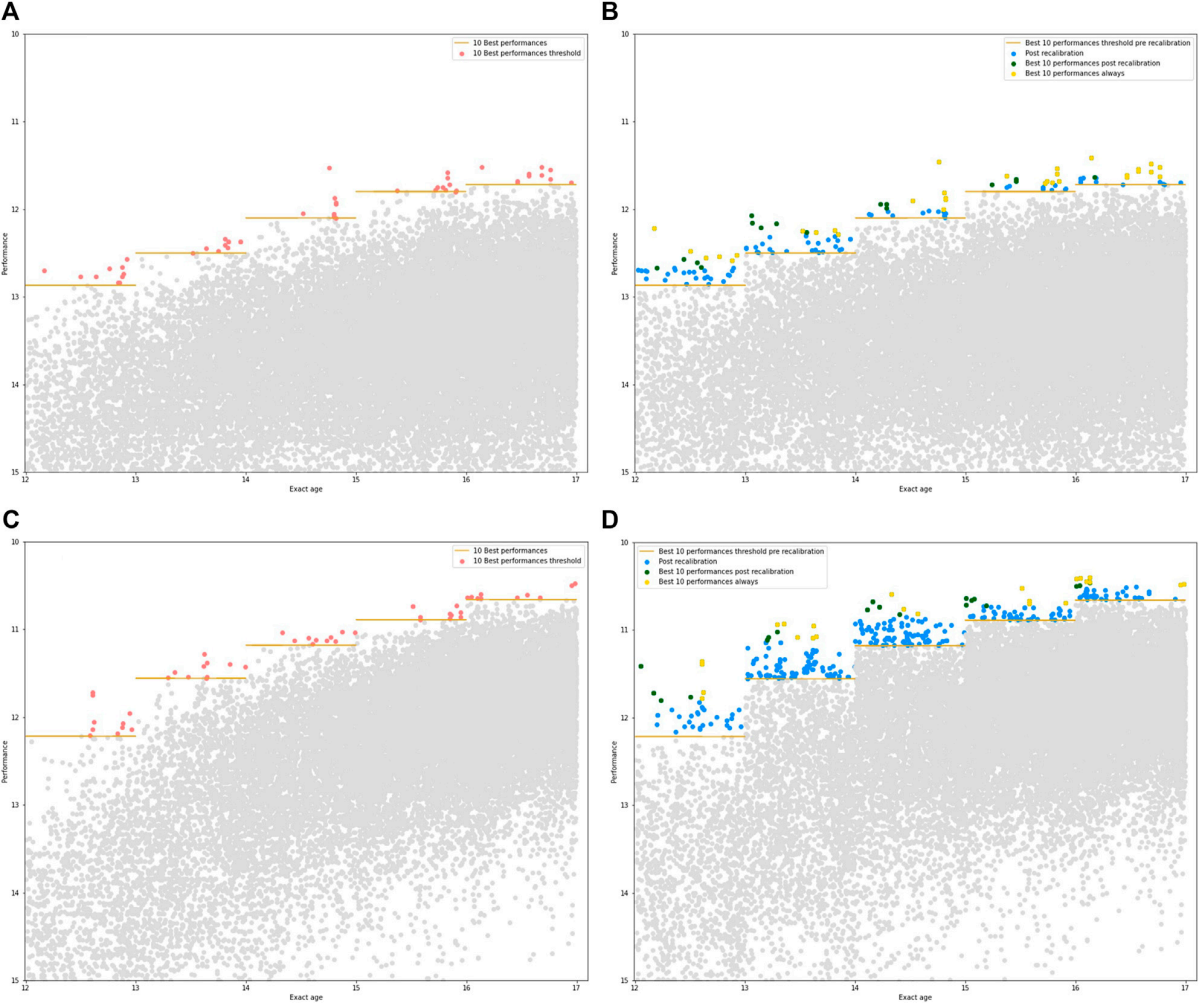
**(A)** Raw time performances among U13–U17 categories of 100 m French female sprinters. Horizontal bars (gold) in each age represent the threshold to reach the 10-Best performances (pink points). **(B)** Time performances post-rebalancing among U13–U17 categories of the same 100 m French female sprinters. Performances above the threshold (but not in the 10-Best pre-rebalancing) after rebalancing are now shown in blue. The performances that were already in the 10-Best before rebalancing are in yellow. The performances that make up to the 10-Best only after rebalancing are colored in green. **(C)** Raw time performances among U13 and U17 categories of 100 m French male sprinters. **(D)** Time performances post-rebalancing among U13–U17 categories of the same 100 m French male sprinters.

The pre-rebalancing performances for females 100 m for U13–U17 categories are shown in Figure 2A. On the age axis, the majority of the 10-Best performances are concentrated towards the end of the age category concerned, meaning that the most performant are the older even in a same-age category.

The rebalanced performances of the same population according to age and the initial performance is presented in Figure 2B. First, new potentials emerge through this rebalancing method. Indeed, there are 35, 37, 23, 18, and 22 of them, respectively at U13, U14, U15, U16, and U17 to exceed the pre-rebalancing 10-Best performances. Then, among them, rebalanced performances come to feed a new 10-Bestr (10-Best rebalanced). In the youth categories, this 10-Bestr is renewed by almost half and more stable with age.

The principle is similar for Figures 2C, D, with pre-rebalancing and post-rebalancing performances for males 100 m for U13–U17 categories respectively. Again, the best performances before rebalancing are achieved by relatively older athletes. On the other hand, more rebalanced performances are above the 10-Best threshold. In the U15 category, 103 new athletes appear in the list, with great potential.

**FIGURE 3:**
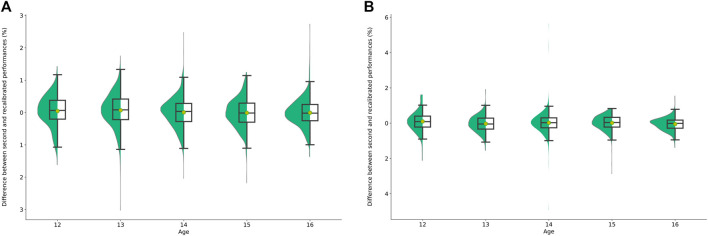
**(A)** Boxplot of percentage differences between rebalanced performances and second performances achieved in the female 100 m of U13–U17 categories. **(B)** Boxplot of percentage differences between rebalanced performances and second performances achieved in the male 100 m of U13–U17 categories.

In order to validate our individual rebalancing method, we compare the rebalanced performance with the second realized performance. The difference between these two performances is shown in Figure 3 in percentage. On average, by category, the rebalanced performance and the second performance achieved are not significantly different. As can be seen, the difference between the two types of performance is centered around zero, so there is no significant difference between the realized performance and the rebalanced performance.

## Discussion

This study shows a strong relative age effect among young French Track and Field athletes. A correcting method is validated to individually rebalance each athlete performance and bring out her/his potential, according to the exact age at the time of performance. Corrections are computed for all events, sexes and age categories.

An asymmetric distribution of birth quarters is shown for U13 and U17 categories among 100 m male sprinters. This RAE increases with the performance level in certain age categories, particularly in the younger ones. RAE was investigated among 77,571 performance data of U13, U15, U17, U20 and senior English athletes (Kearney et al., 2018). They found that U13 male 100 m sprinters were 6.3 times more likely to be born in the first trimester (Q1 vs*.* Q4), an odds ratio that decreased with age. The probability of being born in Q1 among U15, U17, U20 and senior is 4.2, 2.6, 2.1, and 1.8 higher than in Q4, respectively. Among French throwers, the U16 category stands out with a strong over-representation of Q1. This trend was in accordance with a recent study (Kearney et al., 2018), where the OR decreased after the U15. Despite the fact that the age categories include a two-year period, the authors have chosen to focus only on the last year of the age category. In our study, we chose to explore each year of the age group in view of the rebalancing method that allows for the comparison of athletes competing together. Other authors have chosen to also look at athletes in both years of the U18 and U20 age categories. Based on 642 spanish athletes, a stronger RAE was also highlighted, measured by odds-ratio, for male U18 and U20 first year, among a restricted sample (Brazo-Sayavera et al., 2018). Indeed, they analyzed athletes who competed at international level between 2006 and 2014 and considered all events at once. Among 61 males U18 first year athletes involved, none was born in the fourth quarter vs. 39 in the first one. Information about selections in national teams was not provided in this study but the distribution was classified by competitiveness levels. National team members are included in the “Top10%;” e.g., among the U18 “Top10%” of the 110 m hurdles males, 35.8% were born in the first trimester vs*.* 13.3% in the last one.

Our results also reveal a strong RAE among female athletes in the 100 m sprint, throws and jumps, enhanced by the level of performance. However, a study analysing the Swiss Talent Development Program did not find RAE among Track and Field female athletes (Romann and Fuchslocher, 2014). Nevertheless, even if they distinguished two types of competitiveness levels (all teenagers between 10 and 20 years old interested in sport and the Talent Development Program level), they did not investigate RAE by age category and considered all events together. Birth quarter distributions of female athletes were also examined on the international championships Spanish track and field athletes (Brazo-Sayavera et al., 2018) and showed significant differences among those belonging to the first year of each category of age (U18 and U20). In the first year of the U18 category, none was born in Q4 and only one in Q3 among the selected 41 females. Among world-class junior female sprinters, RAE was also found with a larger effect in the higher levels (Brustio and Boccia, 2021). “All,” “Top10,” and “Top50,” 100 m sprinters were 1.7, 1.9, and 2.3 times more likely to be born in Q1 than in Q4. The gap was larger among 400 m sprinters, for “All,” “Top100,” and “Top50,” with an odds-ratio of 1.7, 2.5, and 3.3, respectively.

### Corrective adjustment procedures

By applying a rebalancing method, many individual potentials that may be lost for the future if undetected are revealed. A quadratic function was applied on 16 and 17 years old male and female athletes (Brustio and Boccia, 2021). Using 16 and 17 years as the reference age, they corrected the performances of the 15.01–16.99 and 16.01–17.99 years old athletes, respectively. After correction, they found a smaller or no RAE among 16 year-old athletes. However, a stronger RAE was revealed among corrected performances of 17 year-old athletes, who ran the 100 m and 200 m. Athletes older than the reference age had their performance deteriorated, while the younger ones had their performance improved. It is then worth noticing that this study was conducted on world-class athletes, who had achieved at least five international performances in their career: it is therefore important to consider the competition level.

7761 sprinters (60 m) between the U9 and U16 categories were considered at different performance levels (Romann and Cobley, 2015). After observing that the RAE increased with performance levels, the authors corrected the performances by applying a regression for all age-groups. They found that RAE was not present anymore for “Top10%,” “Top25%,” and “Top50%” excepted for U9 and U10. This study does not investigate the same age range. As the practice of middle- and long-distances comes at later ages than sprints, we focused on earlier ages for sprints and later ages for other distances. A trend confirmed in a study (Marc et al., 2018), that spotlighted the age difference between the peaks of performance in the 100 m and 6-day ultra-marathon (Marc et al., 2018). The male performance peak was reached at 24.7 years on the 100 m and at 38.1 years for ultra-marathon races. The same trend was observed for females, with a performance peak age at 25 on the 100 m and at 43 for the 6-day ultra-marathon (Marc et al., 2018). This study focuses on different age groups with respect to the considered event.

The main purpose of this study was to reveal the true potential of athletes, once the relative age effect was taken into account. Regressions were run in each age group. Coefficients of determination are high (between .62 and .94 for female, between .81 and .95 for males), showing the consistency of the results and the importance of better appreciating athlete’s potentials with respect to her/his age group. It is now necessary to think in terms of age groups and not over the whole spectrum of age categories.

Correction methods have been applied in youth track-and-field, such as in male and female long jump. In those studies, RAE was spotlight among “All,” “Top25%,” and “Top10%” male long jumpers with a greater effect when the level of competitiveness increased. The authors (Brustio et al., 2022) used polynomial regressions of degree 2 to obtain the rebalancing coefficient. After correction, RAE was no longer spotted among Top25% and Top10% male jumpers. In this study, we chose to run a linear regression by age category to better capture the importance of the relative age effect within each age category. The intention was also to rebalance performances and to observe individually whether this rebalancing allowed potential to emerge. Indeed, the aim was to see the individual impact on performance and not the collective impact on the RAE, hence the rebalancing of all performances and not just the best ones. In addition, validation methods also differ. On one side is checked how much the RAE is reduced by rebalancing the performance. On the other side, the rebalancing method is applied individually to a performance in order to observe how close the rebalanced performance is to the realized one. That is why this method is more revealing of young potentials.

Peak performance is reached earlier in females than in males in these younger categories studying here. This may be related to peak growth velocity. Indeed, studies (Tanner et al., 1966; Largo et al., 1978) showed that girls reach their peak growth velocity between the ages of 11 and 13 years, whereas for boys it happens later, between 13 and 15 years. This has an impact on the rebalancing coefficients, which converge more quickly to 0 than for male.

Also, the popularity of an event may have a non-negligeable impact on RAE (Cobley et al., 2009), especially in popular sports. A concept confirmed by another study, that stated that competition is a fundamental condition for the rise of RAE (Musch and Grondin, 2001).

We can also wonder if the popularity of an event does, or not, provide the conditions for a recurence loop, when more media coverage brings more practitioners and higher competitiveness, reinforcing the process of selection. This may explain our non-significant result of skewed distributions of birth quarters in some age categories. RAE is larger in events where speed and strength are emphasised (Hollings et al., 2014). In the French Youth Swimming Championships (U11 up to U19), for example, the number of female swimmers participating to the Freestyle 1500 m is 25 times lower than in Freestyle 50 m (Difernand et al.). This may explain the link between RAE, competitiveness and popularity.

For all the reasons mentioned above, corrective adjustments should be considered in talent identification and detection programs. Indeed, federations, coaches and staff members should be aware that these methods may reduce biases and dropout rates and increase participation and interest among young talented athletes (Abbott et al., 2020).

## Limitations

The main advantage of this study is the large size of the French Athletics Federation database. It contains all the performance data of French athletes since 2009. However, our rebalancing method has some limits. One of them concerns young people who come to try the discipline for a few years without being regular. These athletes who come to try out the discipline without necessarily intending to commit themselves seriously may disrupt the linear regressions and therefore the coefficient that demonstrates the significance of the relative age effect. The coefficient then loses precision and this does not correspond to the evolution of performance (Berthelot et al., 2012; Berthelot et al., 2019). This also leads to a loss of precision in terms of rebalanced performance. Another limitation is the paucity of data in some events, the so-called “late maturation” disciplines, where athletes begin to practice at a late age. For example, the 1500 m race is not popular under the age of 15, making the detection process difficult. As suggested (Delorme et al., 2011) and as shown in a study of young Spanish sportsmen between 9 and 14 years old (Gil et al., 2021), there may be a sport popularity effect that is at the origin of the relative age effect. Indeed, the larger the number of participants, the stronger the RAE, which has been shown at the level of different sports (football, rugby, ice hockey) but we can also imagine that this is the case within a single sport for several events. If there is no relative age effect, then there is no need to rebalance performance. The last limitation is the evolution in rules and regulations based on age category in a particular event. For example, in throw events, the weight of the thrown object increases with age; so is the height of the hurdles in the hurdle race events. Thus, the question of athlete adaptability to rule changes and their impact on performance hierarchy remains open for future research.

## Conclusion

A relative age effect is present in all types of Track and Field events among both males and females. The level of competitiveness enhances relative age effect. Corrective adjustment methods provide a better objectivation of athletes’ performances. Indeed, it is important to make coaches aware of relative age effect. Then, the method we have put in place is designed to be practical for coaches, sport scientists and athletes to apply in the field in order to optimise talent identification and detection programs.

## Data Availability

The datasets presented in this article are not readily available. Indeed, the datasets generated for this study will not be made publicly available because they were provided by the federation in confidentiality. Requests to access the datasets should be directed to IT Service, ffa@athle.fr.

## References

[B1] Abbott S. Hogan C. Castiglioni M. T. Yamauchi G. Mitchell L. J. G. Salter J. (2021). Maturity-related developmental inequalities in age-group swimming: The testing of “Mat-CAPs” for their removal. J. Sci. Med. Sport 24, 397–404. 10.1016/j.jsams.2020.10.003 33172611

[B2] Abbott S. Moulds K. Salter J. Romann M. Edwards L. Cobley S. (2020). Testing the application of corrective adjustment procedures for removal of relative age effects in female youth swimming. J. Sports Sci. 38, 1077–1084. 10.1080/02640414.2020.1741956 32202222

[B3] Berthelot G. Bar-Hen A. Marck A. Foulonneau V. Douady S. Noirez P. (2019). An integrative modeling approach to the age-performance relationship in mammals at the cellular scale. Sci. Rep. 9, 418. 10.1038/s41598-018-36707-3 30674921 PMC6344496

[B4] Berthelot G. Len S. Hellard P. Tafflet M. Guillaume M. Vollmer J.-C. (2012). Exponential growth combined with exponential decline explains lifetime performance evolution in individual and human species. Age (Dordr) 34, 1001–1009. 10.1007/s11357-011-9274-9 21695422 PMC3682058

[B5] Boccia G. Moisè P. Franceschi A. Trova F. Panero D. La Torre A. (2017). Career performance trajectories in track and field jumping events from youth to senior success: The importance of learning and development. PLoS One 12, e0170744. 10.1371/journal.pone.0170744 28129370 PMC5271320

[B6] Brazo-Sayavera J. Martínez-Valencia M. A. Müller L. Andronikos G. Martindale R. J. J. (2018). Relative age effects in international age group championships: A study of Spanish track and field athletes. PLoS One 13, e0196386. 10.1371/journal.pone.0196386 29689117 PMC5916855

[B7] Brustio P. R. Boccia G. (2021). Corrective procedures remove relative age effect from world-class junior sprinters. J. Sports Sci. 39, 2603–2610. 10.1080/02640414.2021.1947618 34210248

[B8] Brustio P. R. Cobley S. Abbott S. La Torre A. Moisè P. Rainoldi A. (2022). Corrective adjustment procedures as a strategy to remove relative age effects: Validation across male and female age-group long jumping. J. Sci. Med. Sport 25, 678–683. 10.1016/j.jsams.2022.04.007 35644757

[B9] Brustio P. R. Kearney P. E. Lupo C. Ungureanu A. N. Mulasso A. Rainoldi A. (2019). Relative age influences performance of world-class track and field athletes even in the adulthood. Front. Psychol. 10, 1395. 10.3389/fpsyg.2019.01395 31275208 PMC6591260

[B10] Cobley S. Abbott S. Eisenhuth J. Salter J. McGregor D. Romann M. (2019). Removing relative age effects from youth swimming: The development and testing of corrective adjustment procedures. J. Sci. Med. Sport 22, 735–740. 10.1016/j.jsams.2018.12.013 30665755

[B11] Cobley S. Baker J. Wattie N. McKenna J. (2009). Annual age-grouping and athlete development: A meta-analytical review of relative age effects in sport. Sports Med. Auckl. N.Z.) 39, 235–256. 10.2165/00007256-200939030-00005 19290678

[B12] Cumming S. P. Brown D. J. Mitchell S. Bunce J. Hunt D. Hedges C. (2018). Premier League academy soccer players’ experiences of competing in a tournament bio-banded for biological maturation. J. Sports Sci. 36, 757–765. 10.1080/02640414.2017.1340656 28628369

[B13] Cumming S. P. Lloyd R. S. Oliver J. Eisenmann J. C. Malina R. M. (2017). Bio-banding in sport: Applications to competition, talent identification, and strength and conditioning of youth athletes. 10.1519/SSC.0000000000000281

[B14] De Larochelambert Q. Difernand A. Antero J. Sedeaud A. Toussaint J.-F. Pierre Yves L. (2022). Relative age effect in French alpine skiing: Problem and solution. J. Sports Sci. 40, 1137–1148. 10.1080/02640414.2022.2052428 35321626

[B15] Delorme N. Chalabaev A. Raspaud M. (2011). Relative age is associated with sport dropout: Evidence from youth categories of French basketball. Scand. J. Med. Sci. Sports 21, 120–128. 10.1111/j.1600-0838.2009.01060.x 20136758

[B16] Difernand A. De Larochelambert Q. Pla R. Barlier K. Marc A. Ferri S. . Corrective adjustment methods for relative age effects on French swimmers’ performances. PLoS One. 10.1371/journal.pone.0283229 PMC10124878 37093823

[B17] Gil S. M. Bidaurrazaga-Letona I. Larruskain J. Esain I. Irazusta J. (2021). The relative age effect in young athletes: A countywide analysis of 9-14-year-old participants in all competitive sports. PLoS One 16, e0254687. 10.1371/journal.pone.0254687 34270609 PMC8284647

[B18] Helsen W. F. Starkes J. L. Van Winckel J. (1998). The influence of relative age on success and dropout in male soccer players. Am. J. Hum. Biol. 10, 791–798. 10.1002/(SICI)1520-6300 2–1. 28561412

[B19] Hollings S. C. Hume P. A. Hopkins W. G. (2014). Relative-age effect on competition outcomes at the world youth and world junior athletics championships. Eur. J. Sport Sci. 14, S456–S461. 10.1080/17461391.2012.713007 24444241

[B20] Kearney P. E. Hayes P. R. Nevill A. (2018). Faster, higher, stronger, older: Relative age effects are most influential during the youngest age grade of track and field athletics in the United Kingdom. J. Sports Sci. 36, 2282–2288. 10.1080/02640414.2018.1449093 29513142

[B21] Largo R. H. Gasser Th. Prader A. Stuetzle W. Huber P. J. (1978). Analysis of the adolescent growth spurt using smoothing spline functions. Ann. Hum. Biol. 5, 421–434. 10.1080/03014467800003071 727700

[B22] Mann D. L. Ginneken P. J. M. A. van (2017). Age-ordered shirt numbering reduces the selection bias associated with the relative age effect. J. Sports Sci. 35, 784–790. 10.1080/02640414.2016.1189588 27238077

[B23] Marc A. Sedeaud A. Schipman J. Saulière G. Toussaint J. F. (2018). Age and performance from 10 seconds to a 6-days race. J. Athl. Enhanc. 07. 10.4172/2324-9080.1000298

[B24] Musch J. Grondin S. (2001). Unequal competition as an impediment to personal development: A review of the relative age effect in sport. Dev. Rev. 21, 147–167. 10.1006/drev.2000.0516

[B25] Roberts S. J. McRobert A. P. Rudd J. Enright K. Reeves M. J. (2021). Research in another un-examined (RAE) context. A chronology of 35 years of relative age effect research in soccer: Is it time to move on? Sci. Med. Footb. 5, 301–309. 10.1080/24733938.2020.1841278 35077305

[B26] Romann M. Cobley S. (2015). Relative age effects in athletic sprinting and corrective adjustments as a solution for their removal. PLoS One 10, e0122988. 10.1371/journal.pone.0122988 25844642 PMC4386815

[B27] Romann M. Fuchslocher J. (2014). The need to consider relative age effects in women’s talent development process. Percept. Mot. Ski. 118, 651–662. 10.2466/30.10.PMS.118k24w8 25068738

[B28] Smith K. L. Weir P. L. Till K. Romann M. Cobley S. (2018). Relative age effects across and within female sport contexts: A systematic review and meta-analysis. Sports Med. 48, 1451–1478. 10.1007/s40279-018-0890-8 29536262

[B29] Tanner J. M. Whitehouse R. H. Takaishi M. (1966). Standards from birth to maturity for height, weight, height velocity, and weight velocity: British children, 1965. I. Arch. Dis. Child. 41, 454–471. 10.1136/adc.41.219.454 5957718 PMC2019592

[B30] Wattie N. Cobley S. Baker J. (2008). Towards a unified understanding of relative age effects. J. Sports Sci. 26, 1403–1409. 10.1080/02640410802233034 18825541

[B31] Williams A. M. Ford P. R. Drust B. (2020). Talent identification and development in soccer since the millennium. J. Sports Sci. 38, 1199–1210. 10.1080/02640414.2020.1766647 32568000

