# Supplementary material for: Relative age effects in track-and-field: Identification and performance rebalancing
Source: Front Physiol. 2023 Jan 12;13:1082174. doi: 10.3389/fphys.2022.1082174 (PMC9879012; doi:10.3389/fphys.2022.1082174)
Supplement: Supplementary file 1 [file Table1.DOCX]

Supplementary Material.

# Supplementary Figures


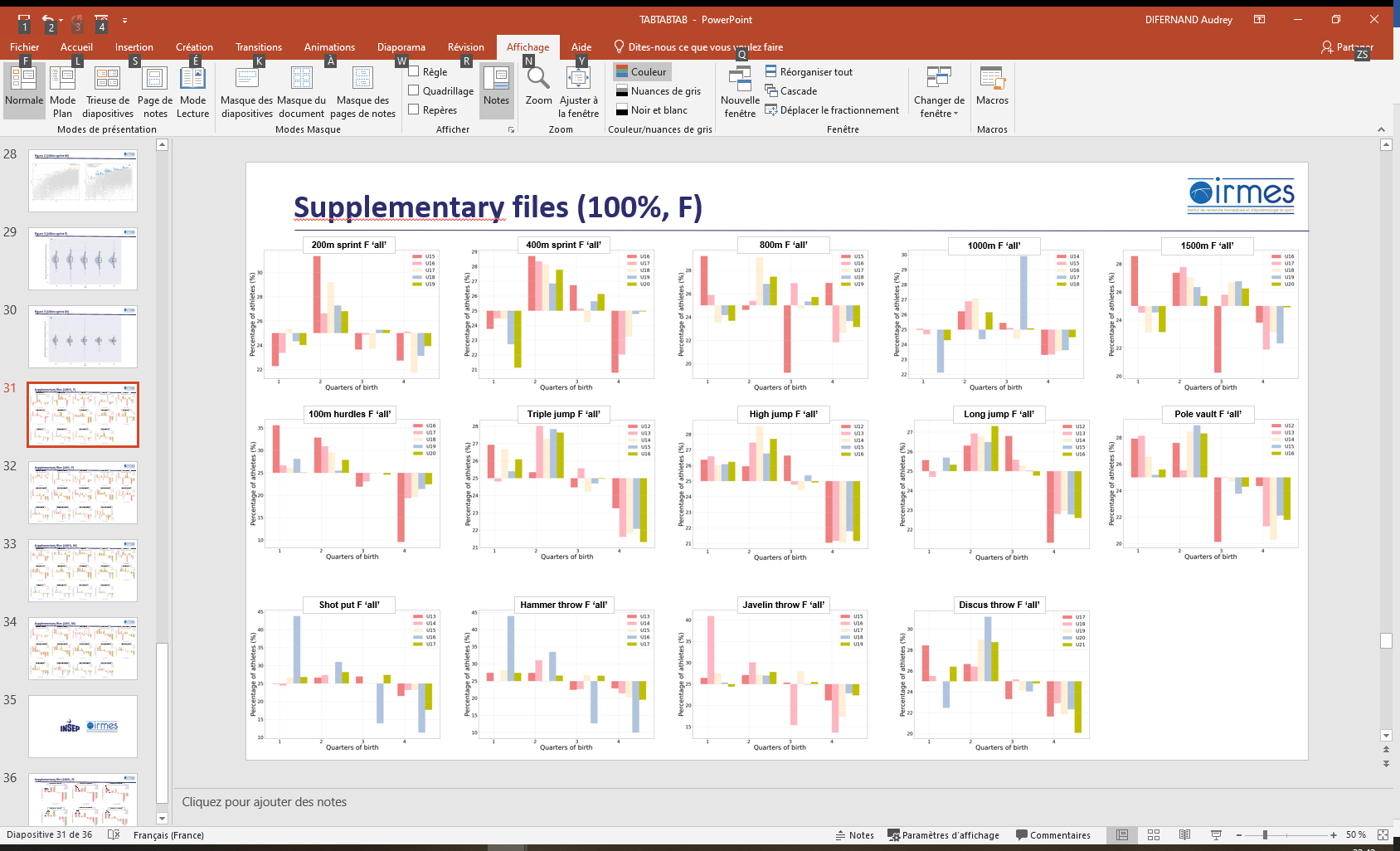


**Supplementary Figure 1A.** Birth quarters distribution according to age categories for ‘all’ female events.


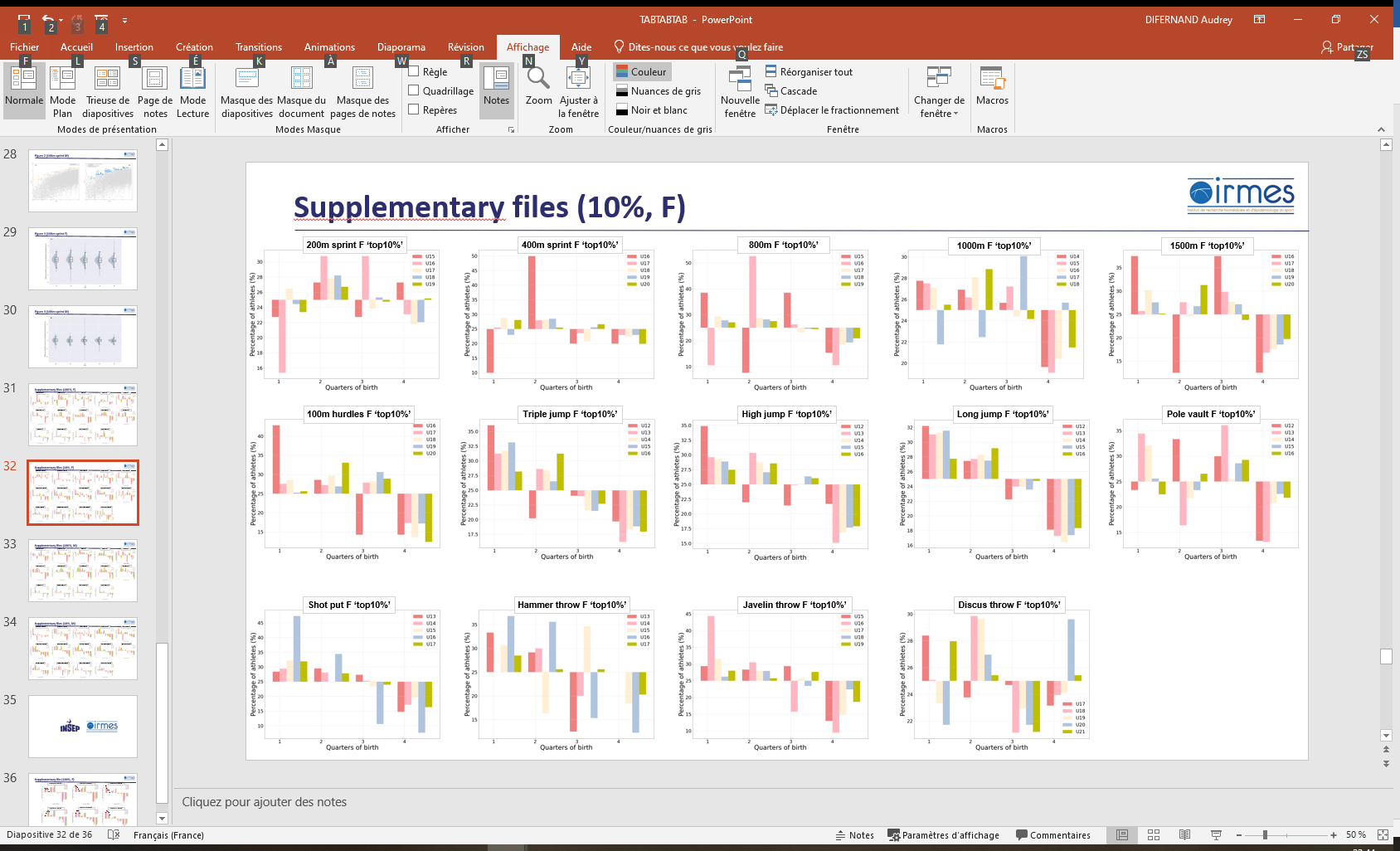


**Supplementary Figure 1B.** Birth quarters distribution according to age categories for ‘Top10%’ female events.


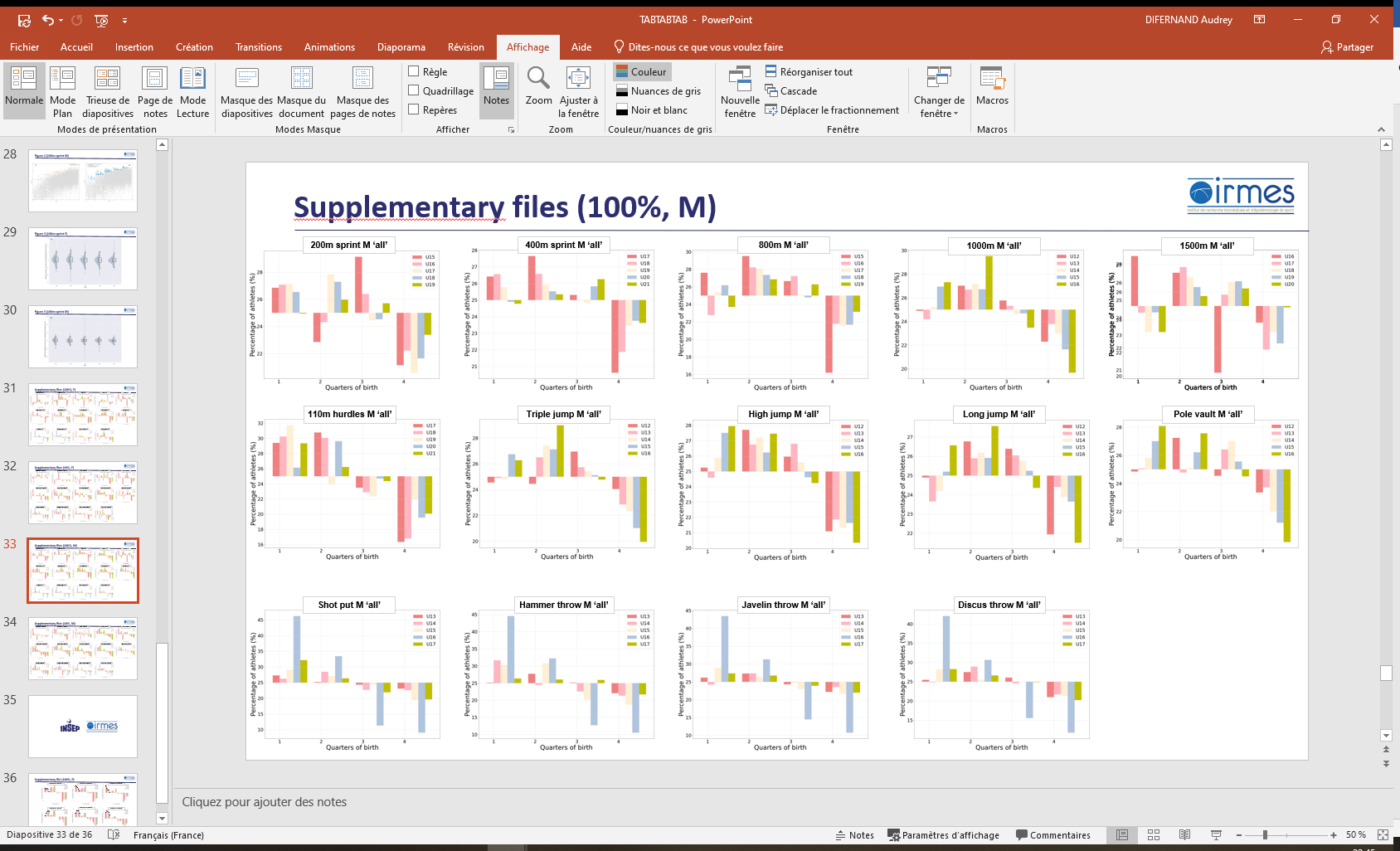


**Supplementary Figure 2A.** Birth quarters distribution according to age categories for ‘all’ male events.


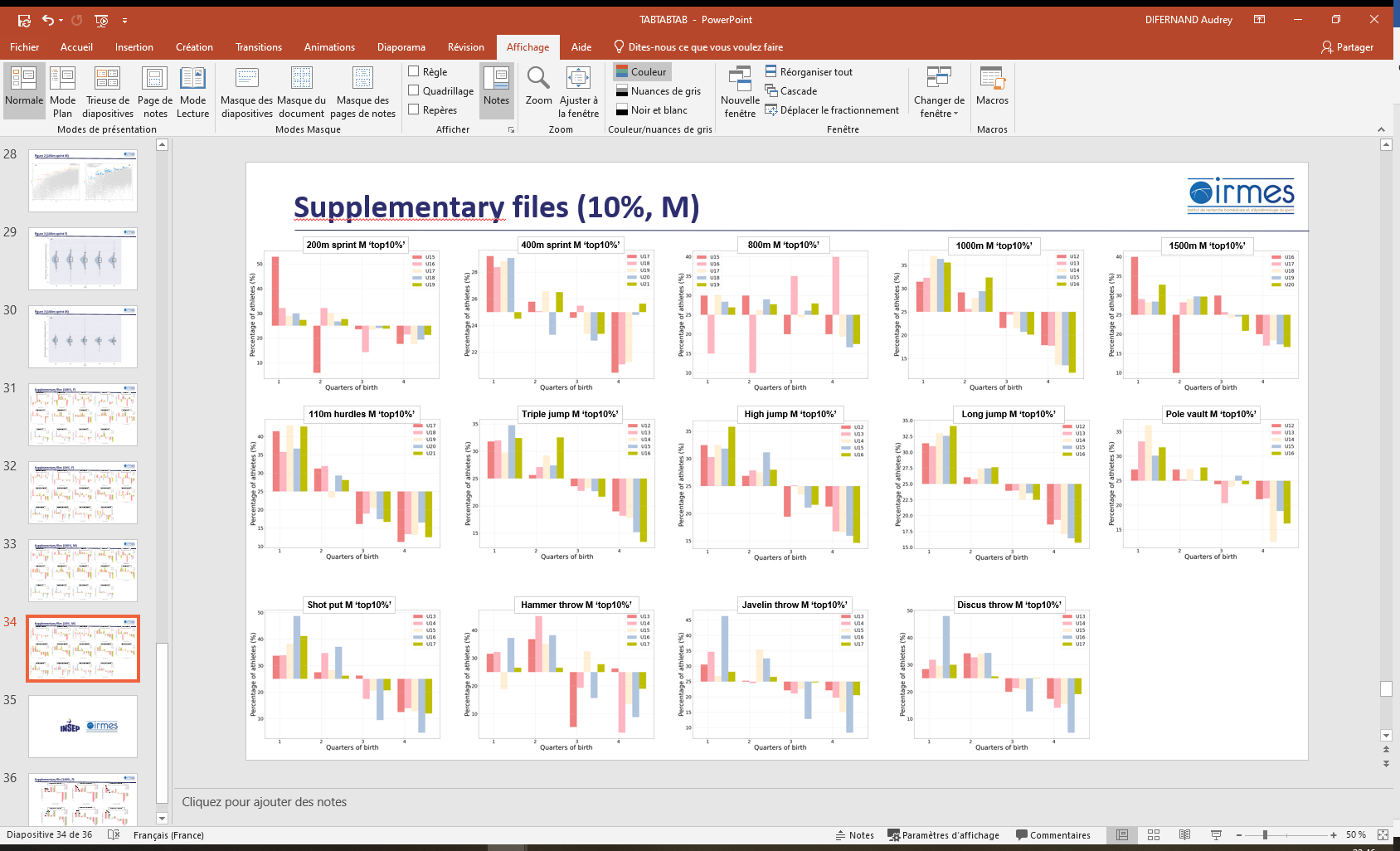


**Supplementary Figure 2B.** Birth quarters distribution according to age categories for ‘Top10%’ male events.
